# Mercury concentration data from Matang Mangrove Forest Reserve, Malaysia

**DOI:** 10.1016/j.dib.2020.105134

**Published:** 2020-01-14

**Authors:** Giovanna Wolswijk, Behara Satyanarayana, Quang Dung Le, Yin Fui Siau, Ahmad Nazila Bin Ali, Ibrahim Sunkanmi Saliu, Muhammad Amir Bin Fisol, Cristina Gonnelli, Farid Dahdouh-Guebas

**Affiliations:** a Systems Ecology and Resource Management, Department of Organism Biology, Faculté des Sciences, Université Libre de Bruxelles (ULB), Avenue F.D. Roosevelt 50, CPi 264/1, B-1050, Bruxelles, Belgium; b Mangrove Research Unit (MARU), Institute of Oceanography and Environment (INOS), Universiti Malaysia Terengganu (UMT), 21030, Kuala Nerus, Terengganu, Malaysia; c Laboratorio di Ecologia e Fisiologia Vegetale, Dipartimento di Biologia, Università degli studi di Firenze (UNIFI), Via Micheli 1, 50121, Firenze, Italy; d Laboratory of General Botany and Nature Management, Biocomplexity Research Focus, Department of Biology, Faculty of Sciences and Bio-engineering Sciences, Vrije Universiteit Brussel (VUB), Campus Oefenplein, VUB-APNA-WE Pleinlaan 2, B-1050, Brussels, Belgium

**Keywords:** Mangrove sediment, Plant tissue, Fauna, Toxic elements, Pollution, *Rhizophora*, Hg partitioning

## Abstract

This paper presents the results of mercury analysis on 786 abiotic (surface sediments) and biotic (plant and animal tissues) samples collected from 10 sites at Matang Mangrove Forest Reserve in Peninsular Malaysia. Sediment samples were collected at the surface level from both river bank and forest understory. Whereas plant tissues obtained from *Rhizophora apiculata* Blume and *Rhizophora mucronata* L. consisted of leaves (in four stages namely young, mature, senescent and decomposing), bark and roots (divided into xylem, cortex and epidermis), the animal samples were represented by muscle tissue of the gastropod *Cassidula aurisfelis* Bruguière and the cockle *Tegillarca granosa* L. The mercury concentration measurements were obtained through a cold vapor atomic absorption spectrometer. The core data have been analysed and interpreted in the paper “Distribution of mercury in sediments, plant and animal tissues in Matang Mangrove Forest Reserve, Malaysia” [1].

**Table:** Specifications Table

Subject	Environmental Science
Specific subject area	Pollution
Type of data	Table
How data were acquired	Cold vapor atomic absorption spectrometer (MA-3000 Nippon Instruments Corp., Japan)
Data format	Raw
Parameters for data collection	1. Surface sediment samples from river bank and understory 2. Plant tissue samples from *Rhizophora apiculata* and *R. mucronata* leaves (young, mature, senescent and decomposing stages), bark and roots (xylem, cortex and epidermis) 3. Animal muscle tissue samples from *Cassidula aurisfelis* and *Tegillarca granosa*
Description of data collection	Both abiotic and biotic samples were collected during June–July 2018 from 10 sites within Kuala Sepetang administrative range of Matang Mangrove Forest Reserve. All biotic samples were first washed with distilled Milli Q water (Millipore Corporation, USA) and then freezedried (−40 °C) (LABCONCO Freeze Dry System/Freezezone 4.5) before grinding to fine powder with mortar and pestle. The sediment samples were subjected to manual sieving with 60 μm mesh size. Grounded samples were finally analyzed for mercury concentration with the help of a cold vapor atomic absorption spectrometer (MA-3000 Nippon Instruments Corp., Japan).
Data source location	Kuala Sepetang, Matang Mangrove Forest Reserve, Perak, Malaysia Station 1: N04°50′36.6″, E100°38′02.1″ Station 2: N04°50′19.2″, E100°37′05.8″ Station 3: N04°50′24.7″, E100°35′38.9″ Station 4: N04°51′09.4'', E100°33′24.6″ Station 5: N04°49′15.1'', E100°35′14.8″ Station 6: N04°49′32.2″, E100°33′39.4″ Station 7: N04°48′56.4'', E100°37′19.2″ Station 8: N04°47′25.3'', E100°37′34.6″ Station 9: N04°47′59.9″ E100°38′41.8″ Station 10: N04°45′46.7″E100°36′18.0″ Cockle culture: N04°51′29.4″, E100°34′43.6″
Data accessibility	With the article
Related research article	Giovanna Wolswijk, Behara Satyanarayana, Le Quang Dung, Yin Fui Siau, Ahmad Nazila Bin Ali, Ibrahim Sunkanmi Saliu, Muhammad Amir Bin Fisol, Cristina Gonnelli, Farid Dahdouh-Guebas, 2019, Distribution of Mercury in sediments, plant and animal tissues in Matang Mangrove Forest Reserve, Malaysia. *Journal of Hazardous Materials*, https://doi.org/10.1016/j.jhazmat.2019.121665.

**Table:** 

**Value of the Data** • The data represents, for the first time, an in-depth analysis of Hg pollution at Matang mangroves in Peninsular Malaysia. The large sample size (n = 786) allows to get reliable information on the distribution of mercury in *Rhizophora* spp. • The data is beneficial to a wider scientific community as few detailed investigations are available on the subject, making this study a benchmark for future research. In addition, the data enables the scientific and local management community to understand the levels of mercury pollution in one of the longest silviculturally managed mangrove forests in the world. • At a local scale, the data can help to take necessary measures for controlling/monitoring the pollution (especially of industrial origin) by concerned authorities like National Hydrological Research Institute of Malaysia, Department of Irrigation and Drainage, Forestry Department, etc. • The present data can be a strong baseline for future studies to define mercury pathways in the mangroves. In addition, the data can be compared with other toxic elements and offer appropriate safety guidelines for the environment as well as the public. • The collection of 786 samples requires time (sample collection and preparation) and energy (manpower and analyses) that can be saved in the planning for future studies on mercury in Matang by other scientists.

## 1 Data description

The data reported in Table 1 consists of raw data on mercury concentrations (n = 786) obtained from 10 sampling sites at Matang Mangrove Forest Reserve in Peninsular Malaysia, that were analyzed and discussed in the study by Wolswijk et al. [1]. The data are subdivided according to the sampling sites (St 1 to St 10). The Hg concentrations in plant tissues are from *R. apiculata* for all sampling sites except St 4 and St 6 (located seaward side) where *R. mucronata* was collected. The value of mercury concentration in sediments collected from the riverbank and inside the forest is the result of the analysis of 5 replicates each. For mangrove leaves we used 10 replicates for each of the four stages (young, mature, senescent and decomposing) considered, and for bark and root samples 6 replicates. For the xylem tissue, the measurements were repeated twice due to difficulty in obtaining a fine powder from the sample grinding. The gastropod - *Cassidula aurisfelis* samples were analysed in 6 replicates per station (found in St 1 to St 6). The measurement of Hg concentration of 10 samples of the mangrove cockle *Tegillarca granosa*, collected from a cockle culture farm in Sangga Besar River, are reported in Table 2. The data accuracy assessment through recovery of the certified reference materials (CRMs) is reported in Table 3.

**Table 1:** Station-wise raw data of mercury concentrations in Matang Mangrove Forest Reserve. All values reported are in μg Kg^−1^. SED IN: sediments from inside the forest (10–15 m from fringe), SED RB: sediments from the river bank, YL: young leaves, ML: mature leaves, SL: senescent leaves, DL: decomposing leaves, B: bark, RE: root epidermis, RC: root cortex, RX: root xylem, G: gastropods. Plant tissue data from St 4 and 6 are from *Rhizophora mucronata*, while others from *R. apiculata*.

Station 1										

SED IN	SED RB	Y L	M L	S L	D L	B	R E	R C	R X	G

42.628	44.822	4.467	18.323	36.898	29.871	0.591	1.196	−1.489	−0.803	71.749
42.082	44.444	10.525	8.391	26.436	38.146	0.34	3.29	−0.94	−1.06	78.019
65.605	45.035	1.137	15.827	38.939	35.288	0.739	3.41	−1.459	−1.915	79.558
44.829	41.623	5.482	14.79	32.599	38.232	−0.248	2.808	−1.051	−1.384	73.81
41.658	44.23	9.766	15.134	33.671	39.864	0.053	3.686	−0.66	−0.963	73.822
		0.426	15.745	27.197	33.88	0.223	0.051	−1.203	−1.233	61.218
		3.41	10.778	30.112	31.132				−0.765	
		2.024	10.558	27.408	37.615				−1.104	
		4.476	6.102	34.795	41.447				−0.602	
		1.35	12.354	27.355	38.324				−0.974	
									−0.598	
									−1.272	
Station 2										
SED IN	SED RB	Y L	M L	S L	D L	B	R E	R C	R X	G
40.903	89.505	1.068	13.333	32.687	40.641	0.033	0.986	−0.779	−0.382	29.234
43.821	87.88	1.922	11.366	38.435	47.107	0.022	1.185	−0.449	−0.793	29.956
42.693	86.406	1.672	15.73	33.484	24.155	4.389	2.389	−0.379	−0.145	32.056
40.881	45.951	0.258	16.203	49.941	38.43	−0.021	2.492	−0.384	−0.37	40.046
42.942	86.228	10.676	20.617	38.41	43.305	2.469	0.576	−0.508	0.503	38.578
		0.052	13.659	36.334	49.33	−0.346	1.045	−0.304	−0.116	34.749
		3.255	15.987	36.747	48.234				−0.573	
		3.302	16.814	36.852	48.152				−0.491	
		1.633	13.936	48.204	40.995				−0.549	
		8.326	9.23	34.093	32.595				−0.629	
									0.05	
									−0.217	
Station 3										
SED IN	SED RB	Y L	M L	S L	D L	B	R E	R C	R X	G
47.861	56.465	4.824	31.949	30.808	31.302	2.665	2.128	−0.19	−0.33	92.709
44.646	42.661	20.407	21.17	33.554	29.869	4.091	8.16	1.453	−0.136	111.68
42.673	46.981	12.049	28.872	30.213	43.88	2.18	7.473	0.15	0.169	167.736
40.099	74.142	14.459	16.126	29.656	33.004	0.11	5.657	1.105	0.108	152.453
46.343	44.081	4.868	20.628	28.723	37.881	1.097	5.835	−0.237	−0.413	110.125
		16.046	18.603	33.635	44.322	1.214	2.308	0.294	−0.074	137.761
		19.176	29.917	29.708	41.138				0.256	
		8.077	30.685	29.305	37.617				−0.026	
		25.286	21.721	40.806	37.032				−0.202	
		8.865	21.882	26.777	39.632				−0.438	
									−0.151	
									−0.349	
Station 4										
SED IN	SED RB	Y L	M L	S L	D L	B	R E	R C	R X	G
40.653	32.564	1.295	26.058	34.645	28.626	3.307	2.599	0.323	−0.099	132.42
44.942	37.352	1.518	29.842	21.698	41.636	1.128	3.82	2.039	−0.055	135.116
34.338	35.603	0.045	11.846	29.708	36.499	2.276	2.568	1.801	0.291	130.605
42.017	33.556	1.614	29.737	29.524	28.858	1.353	3.2	−0.183	0.1	123.409
37.464	30.801	1.26	28.915	32.59	37.018	2.145	2.85	0.151	−0.159	117.218
		0.427	22.412	27.647	41.414	0.606	1.09		−0.052	111.508
		7.252	20.834	46.91	37.354				−0.262	
		1.206	23.323	33.544	36.842				−0.251	
		1.36	27.433	24.877	30.279				0.754	
		−0.243	22.003	35.698	31.95				0.833	
									−0.162	
									−0.449	
Station 5										
SED IN	SED RB	Y L	M L	S L	D L	B	R E	R C	R X	G
38.532	40.057	2.918	26.581	29.12	26.996	0.42	1.331	0.022	−0.345	134.8
39.762	39.259	13.927	37.686	29.806	28.616	1.684	4.114	0.228	−0.508	125.205
44.659	35.447	0.252	24.685	32.569	30.867	0.793	2.898	1.056	−0.317	121.025
37.219	36.221	7.863	25.426	33.308	30.264	0.462	1.943	−0.056	−0.127	137.778
33.571	42.223	2.785	28.124	32.243	33.849	5.009	3.529	0.622	−0.075	118.955
		1.063	33.307	33.356	37.279	8.279	2.577	1.134	0.143	104.009
		2.374	26.246	32.609	32.383				−0.069	
		0.335	42.837	35.145	32.504				−0.327	
		1.514	23.921	39.373	30.607				−0.095	
		3.938	34.594	35.986	30.284				−0.179	
									−0.171	
									−0.145	
Station 6										
SED IN	SED RB	Y L	M L	S L	D L	B	R E	R C	R X	G
37.585	35.168	5.207	18.363	31.914	35.05	4.759	2.574	−0.383	−0.432	113.123
40.816	36.771	4.275	31.057	33.038	37.245	1.286	2.597	0.397	−0.289	116.73
42.573	36.991	3.998	18.671	34.218	37.139	0.602	1.388	0.514	−0.278	140.402
34.687	34.889	4.105	15.987	30.529	35.451	4.467	1.371	2.119	−0.332	107.305
34.936	36.834	2.706	12.754	28.549	35.459	2.869	2.289	0.782	0.421	113.863
		2.315	22.678	32.38	25.737	0.395	3.251	0.356	0.133	109.587
		1.741	17.67	39.065	33.591				−0.08	108.821
		4.194	10.49	32.528	32.932				−0.279	
		3.601	19.012	31.187	39.434				−0.146	
		4.385	17.743	34.89	34.962				−0.048	
									−0.391	
									−0.462	
Station 7										
SED IN	SED RB	Y L	M L	S L	D L	B	R E	R C	R X	
56.374	63.69	0.235	5.166	32.202	26.633	−0.199	1.027	−0.264	−0.248	
58.7	47.589	0.136	12.446	51.04	47.988	1.23	1.163	−0.582	−0.269	
56.373	77.462	−0.145	20.864	44.751	19.213	0.404	2.996	−0.483	−0.448	
53.822	50.594	0.097	23.369	39.552	41.734	0.048	3.079	−0.186	−0.573	
54.824	48.632	−0.068	12.36	45.446	31.898	0.37	2.581	−0.534	−0.415	
		0.123	23.54	53.526	34.898	−0.061	2.24	−0.292	−0.523	
		0.056	16.185	36.433	37.452				−0.138	
		−0.247	9.109	38.989	39.819				−0.398	
		−0.291	7.699	49.666	42.398				−0.526	
		0.367	13.563	34.337	30.71				−0.513	
									−0.307	
									−0.573	
Station 8										
SED IN	SED RB	Y L	M L	S L	D L	B	R E	R C	R X	
50.126	48.238	2.259	11.07	34.963	37.181	0.866	3.903	−0.136	−0.473	
43.415	41.013	0.84	16.161	34.704	37.638	1.97	3.92	0.171	−0.723	
50.673	41.702	0.796	17.94	31.44	28.07	0.604	5.876	0.507	−0.19	
48.037	44.078	0.772	14.317	30.833	23.539	1.445	5.291	1.488	0.169	
48.101	48.775	1.548	14.847	37.002	37.134	1.535	8.708	0.429	0.011	
		4.049	5.771	29.501	45.792	1.507	1.933	−0.224	−0.043	
		0.632	14.763	23.503	36.811				0.227	
		0.391	13.634	37.898	35.386				0.356	
		0.743	7.351	23.349	42.164				0.123	
		0.655	12.796	34.852	33.908				−0.061	
									−0.265	
									−0.242	
Station 9										
SED IN	SED RB	Y L	M L	S L	D L	B	R E	R C	R X	
71.165	64.784	−0.174	21.396	27.32	48.585	2.493	3.747	−0.188	−0.478	
68.157	59.296	−0.165	12.983	32.734	19.73	0.48	4.436	0.386	−0.337	
58.737	48.058	−0.547	13.697	35.701	33.731	0.843	7.813	−0.545	−0.213	
71.934	56.894	−0.544	21.215	28.68	34.918	0.323	5.537	−0.195	−0.32	
66.949	67.209	0.531	14.851	30.713	41.228	0.029	5.9	−0.432	−0.606	
		0.275	17.5	34.67	36.439	0.657	3.502	−0.289	−0.601	
		0.11	23.122	28.09	35.178				−0.29	
		1.303	25.673	31.174	38				−0.371	
		−0.327	11.116	36.794	32.839				−0.374	
		0.023	19.113	27.952	31.446				−0.472	
									−0.352	
									−0.401	
Station 10										
SED IN	SED RB	Y L	M L	S L	D L	B	R E	R C	R X	
56.485	45.495	0.633	16.077	39.552	44.158	0.464	2.165	−0.506	−0.474	
81.203	43.121	0.078	29.665	34.291	31.068	0.358	2.898	−0.407	−0.376	
76.61	84.061	−0.099	36.64	32.969	48.354	2.945	1.426	−0.304	−0.32	
82.962	73.026	0.45	27.571	35.381	31.907	0.309	1.814	−0.407	−0.498	
79.519	40.92	1.174	23.599	33.94	34.561	0.178	2.928	−0.36	−0.579	
		0.487	11.619	35.554	37.925	1.077	2.582	−0.36	−0.586	
		0.329	13.381	37.046	41.622				−0.463	
		−0.055	20.568	34.96	41.686				−0.387	
		0.485	22.94	27.035	33.962				−0.462	
		1.336	28.614	27.529	32.838				−0.653	
									−0.493	
									−0.345

**Table 2:** Mercury concentration in mangrove cockles - *Tegillarca granosa* L. collected from Matang Mangrove Forest Reserve (raw data from 10 replicates) (S = sample).

S1	S2	S3	S4	S5	S6	S7	S8	S9	S10
33.636	29.333	25.37	32.282	21.971	25.249	31.333	29.046	26.267	21.477

**Table 3:** Data accuracy assessment. Recovery percentage of the CRM NIST-SRM 2976 (freeze-dried mussel tissue) and CRM NIST-SRM2702 (marine sediments).

date	measured value	certified value	recovery %
SRM 2976			
August 02, 2018	59.576	61	97.7
August 05, 2018	58.615	61	96.1
August 06, 2018	57.989	61	95.1
August 07, 2018	62.595	61	102.6
August 08, 2018	63.942	61	104.8
August 09, 2018	59.884	61	98.2
August 12, 2018	57.097	61	93.6
August 13, 2018	60.917	61	99.9
August 14, 2018	52.913	61	86.7
August 15, 2018	55.391	61	90.8
August 16, 2018	57.130	61	93.7
August 17, 2018	57.891	61	94.9
August 19, 2018	58.092	61	95.2
August 20, 2018	49.939	61	81.9
August 20, 2018	53.039	61	86.9
SRM2702			
July 03, 2018	337.381	447.4	75.4
July 03, 2018	455.431	447.4	101.8
July 04, 2018	421.724	447.4	94.3
July 05, 2018	382.092	447.4	85.4
August 17, 2018	568.737	447.4	127.1
August 17, 2018	425.107	447.4	95.0

## 2 Experimental design, materials, and methods

At each sampling station, surface sediments (upper 2–5 cm) were collected (with a hand shovel) from both the riverbank (at the water edge) and the inside of the mangrove forest (10–15 m) in 10 replicates, at a distance of 3–5 m following a linear geometry. For the plant tissues, leaves and roots were collected from *Rhizophora apiculata* in all stations except for St 4 and 6, where *R. mucronata* was abundant instead. Samples were taken from ten randomly chosen adult trees inside the forest. Leaf samples were collected in relation to the young, mature, senescent and decomposing stages. Ten replicates were taken per leaf stage per site. Young and mature leaves were hand-collected from the trees, while senescent and decomposing leaves were collected from the forest floor. Root and bark samples were collected (six replicates per station from six different trees) using a knife. Small roots near the sediment surface were targeted for the sampling. The specimens of mangrove gastropod - *Cassidula aurisfelis* were collected manually under the trees selected for plant tissues sampling (St 1 to 6). The edible and economically important mangrove cockles - *Tegillarca granosa* were collected from a cockle culture area in Sangga Besar river. All samples were placed in labeled polythene zip-lock covers and kept in an icebox before transferring to the laboratory for further preservation and analyses.

At the Institute of the Institute of Oceanography and Environment (INOS) laboratory (Universiti Malaysia Terengganu-UMT), sediment samples were put into 15 ml test tubes with a spatula. Samples other than sediments were carefully washed with tap water and then with distilled Milli-Q water (Millipore Corporation, USA) to remove the debris. After washing, 2–6 leaves were pooled together and wrapped in sterile aluminium foil (that was put in furnace at 260 °C for 1 hour to avoid any Hg contamination). Roots were cut with a steel knife and three different tissues were separated per each root sample: epidermis, cortex and xylem. Samples were cut into small pieces and put in 15 ml test tubes.

For gastropods and cockles, the muscle tissue was gently extracted from the shell with aid of tweezers and separated from the visceral tissue. Three gastropods were pooled together in order to get enough material to perform the Hg analysis (for a total of six replicates per station). In the case of cockles two individuals were pooled to make one sample and ten replicates were made. Afterwards the samples were put in 15 ml tubes. For the handling of gastropods and cockles, ethical approval was obtained by the Ethical Biosecurity Committee of the INOS, UMT.

For the drying process all samples were kept in a deep freezer at −80 °C for 48 hours and subsequently put in a freeze dryer (LABCONCO Freeze Dry System/Freezezone 4.5) with pressure lower than 0.133 mBar and temperature of −40 °C for 48–72h. Sediments samples were grinded to fine powder with mortar and pestle, then sieved with 60 μm mesh size, to get homogeneous samples and to separate the sediment particles from other materials (*e.g.* plant debris). Leaf, root and mollusc samples were grinded with mortar and pestle till a fine powder was obtained. For the xylem samples, it was not possible to get a homogeneous result, so two Hg measurements per sample were taken to validate the data (Table 1). Total Hg concentration was measured with a direct mercury analyser (MA-3000, Nippon Instruments Corporation) with detection limit of 0.02 ng of total Hg. Measurements were done at wavelength of 253.7 nm. Prior to analysis, a calibration curve was made with seven Hg standards (STD) with Hg content from 0 to 100 ng (namely 0, 5, 10, 15, 20, 50 and 100 ng). Linear regression was done with the function “lm” in R software, multiple R^2^ was equal to 0.9961 and the p-value was 3.145 × 10^−7^.

For the accuracy assessment of the measurements, a STD solution of 0.1 ppb covered with additive B (Nippon Instrument Corporation) and certified reference materials (CRM) were run before and after the samples. For plant tissues and mollusks, the CRM NIST-SRM2976 (freeze-dried mussel tissue) with a concentration of 61.0 (±3.6) μg Kg^−1^ was chosen, whereas for sediments the CRM NIST-SRM2702 (marine sediments) with a concentration of 447.4 (±6.9) μg Kg^−1^ was used.
